# Abortion care at 20 weeks and over in Victoria: a thematic analysis of healthcare providers’ experiences

**DOI:** 10.1186/s12884-024-06299-0

**Published:** 2024-02-06

**Authors:** Mary Malek, Caroline SE Homer, Clare McDonald, Catherine M Hannon, Paddy Moore, Alyce N Wilson

**Affiliations:** 1 Monash University, Wellington Rd, Clayton, VIC 3800 Australia; 2 Burnet Institute, 85 Commercial Rd, Melbourne, VIC 3004 Australia; 3 Royal Women’s Hospital, 20 Flemington Rd, Parkville, VIC 3052 Australia

**Keywords:** Abortion, Termination, Abortion provider, Abortion access, Quality care, Thematic analysis

## Abstract

**Background:**

In many countries, abortions at 20 weeks and over for indications other than fetal or maternal medicine are difficult to access due to legal restrictions and limited availability of services. The Abortion and Contraception Service at the Royal Women’s Hospital in Victoria, Australia is the only service in the state that provides this service. The views and experiences of these abortion providers can give insight into the experiences of staff and women and the abortion system accessibility. The aim of this study was to examine health providers’ perceptions and experiences of providing abortion care at 20 weeks and over for indications other than fetal or maternal medicine, as well as enablers and barriers to this care and how quality of care could be improved in one hospital in Victoria, Australia.

**Methods:**

A qualitative study was conducted at the Abortion and Contraception Service at the Royal Women’s Hospital. Participants were recruited by convenience and purposive sampling. Semi-structured interviews were conducted one-on-one with participants either online or in-person. A reflexive thematic analysis was performed.

**Results:**

In total, 17 healthcare providers from medicine, nursing, midwifery, social work and Aboriginal clinical health backgrounds participated in the study. Ultimately, three themes were identified: ‘Being committed to quality care: taking a holistic approach’, ‘Surmounting challenges: being an abortion provider is difficult’, and ‘Meeting external roadblocks: deficiencies in the wider healthcare system’. Participants felt well-supported by their team to provide person-centred and holistic care, while facing the emotional and ethical challenges of their role. The limited abortion workforce capacity in the wider healthcare system was perceived to compromise equitable access to care.

**Conclusions:**

Providers of abortion at 20 weeks and over for non-medicalised indications encounter systemic enablers and barriers to delivering care at personal, service delivery and healthcare levels. There is an urgent need for supportive policies and frameworks to strengthen and support the abortion provider workforce and expand provision of affordable, acceptable and accessible abortions at 20 weeks and over in Victoria and in Australia more broadly.

**Supplementary Information:**

The online version contains supplementary material available at 10.1186/s12884-024-06299-0.

## Background

Universal access to safe abortion is fundamental to upholding sexual and reproductive health and rights, and a key part of attaining the United Nations Sustainable Development Goals (SDGs) for good health and well-being (SDG3) and gender equality (SDG5) [1]. When a woman or pregnant person^1^ is unable to access a safe abortion, they may resort to unsafe abortion. It is estimated that 45% of abortions worldwide are performed in unsafe circumstances and represent a major preventable cause of maternal morbidity and mortality [1].

Abortions at 20 weeks’ gestation and over that are sought for indications other than fetal or maternal medicine (also referred to as ‘non-medicalised’ or ‘psychosocial’ indications in this paper) are highly stigmatised and associated with particular access difficulties due to legal restrictions and a limited availability of services [1, 2]. Abortions at this gestation are technically more complex than earlier abortions and have a greater risk of complications [1, 3]. The reasons for seeking an abortion are complex and multifaceted [4, 5]. Non-medicalised reasons for seeking an abortion at any gestational age may include socioeconomic circumstances, a desire to delay or limit childbearing, partner-related reasons, family violence, sexual assault and/or mental illness [4, 6]. Abortions may be delayed until 20 weeks’ gestation due to a delay in discovering the pregnancy, difficulty deciding on an abortion and/or finding and accessing an abortion provider, all of which can be influenced by a woman or pregnant person’s personal circumstances and the context in which they are living [7–10].

In Victoria, Australia, abortion was legalised in 2008 and is available on request until 24 weeks’ gestation, after which the approval of two medical practitioners of any kind is needed to proceed [11]. However, access to abortion at 20 weeks and over in Victoria is complicated by the scarcity of services and providers who perform the procedure and institutional barriers that can delay care [12]. The Abortion and Contraception Service (ACS) at the Royal Women’s Hospital, a public hospital in metropolitan Melbourne, is the only service in the state that provides abortions at 20 weeks and over for indications other than fetal or maternal medicine.

There is little research exploring the care experiences of abortion providers catering for women and pregnant people choosing abortion at 20 weeks and over for psychosocial reasons, and none in the Australian context. For example, a study undertaken in New Zealand found that midwives providing abortions at 20 weeks and over found their work emotionally burdensome and felt inadequately supported [13]. There are few other studies addressing this specific area of care. Investigating abortion providers’ care experiences can generate insight into the care that is delivered, identify strengths and areas for improvement in abortion practice, and inform interventions and policies to improve care at the user and health system levels.

The aim of this study was to examine health providers’ perceptions and experiences of providing abortion care at 20 weeks and over for indications other than fetal or maternal medicine, as well as enablers and barriers to this care and how quality of care could be improved in one hospital in Victoria, Australia.

## Methods

### Study design and setting

A qualitative study design was undertaken to gain an in-depth understanding of the views and experiences of healthcare providers who perform abortions at 20 weeks and over for women’s psychosocial reasons. The study was set at the Abortion and Contraception Service at the Royal Women’s Hospital, a public specialist women’s hospital in metropolitan Melbourne, Victoria catering for more than 9000 births per year [14]. The ACS is the only service providing abortions at 20 weeks and over for non-medicalised reasons in Victoria. Its workforce consists of a multidisciplinary team of obstetrician-gynaecologists, nurses, midwives, social workers and other healthcare providers. Approximately 12 abortions at 20 weeks and over are conducted each month at the service, and at least one abortion over 24 weeks’ gestation each month, for indications other than fetal or maternal medicine. The ACS provides surgical abortions for any indication up to 24 weeks’ gestation. After 24 weeks an induction of labour is performed, and patients are cared for by multiple units and services that includes the ACS.

### Participants and recruitment

Participants were healthcare providers of any discipline aged 18 years and over who are regularly involved in the care of patients having an abortion at 20 weeks and over for indications other than fetal or maternal medicine^2^. Participants were initially recruited by convenience sampling, after which initial interviews were conducted with key personnel in the clinic, including doctors, social workers and nurses. Purposive sampling was then employed to ensure that all disciplines and levels of experience within the ACS were adequately captured, including staff members who care for patients having an abortion at 20 weeks and over but do not directly work in the ACS clinic, such as theatre nurses. Participants were contacted for recruitment by email or approached in-person by members of the research team (CMD, CMH) who are also ACS staff members.

### Data collection

Semi-structured interviews were conducted by members of the research team who are also experienced ACS providers, including a nurse/midwife and social workers (CMH, CMD). There was a general structure to the interview, but this also enabled participants to explore tangents and personal areas of interest. Interviewers were experienced in reflective listening and trauma-informed care and were able to identify and provide support to participants in distress, guided by a distress protocol. None of the participants became distressed during the interviews. A follow-up call was also undertaken one week after the interview to check on participant health and wellbeing.

The interview questions focussed on providers’ care experiences and barriers and facilitators to providing quality abortion care (see Additional file 1). The interview guide was reviewed and revised several times by the research team before being used. Interviews were conducted from April 2022 to December 2022, either in-person in a private and mutually convenient location or via online video conferencing (Zoom software), at a time convenient to both the participant and researcher. A single interview was conducted with each participant. Participants were reimbursed with a $50 Coles/Myer (shopping) voucher for their time. Interviews were recorded using a handheld recording device or the record function on Zoom, depending on the interview format. Field notes were taken by researchers and cross-checked with participants following interviews, there was no further follow up with participants.

### Data analysis

Interviews were transcribed verbatim using Otter transcription software [15]. MM manually edited and reviewed the transcripts for accuracy whilst listening to the audio recordings. A study identification number (i.e., P1, P2, et cetera) was assigned to each interview and all identifying markers were manually removed from the transcripts to maintain confidentiality and privacy. Reflexive thematic analysis was performed using Braun and Clarke’s approach [16], as it allowed for the inductive development of themes to produce novel insights into this under-researched topic. Data analysis was managed using NVivo software (Version 1.6.1).

Reflexive thematic analysis was primarily conducted by MM using the six recursive steps: familiarisation, coding, generating initial themes, reviewing and developing themes, refining, defining and naming themes, and writing up [16]. The first stage of the analysis involved familiarisation with the transcripts, which was achieved by listening to audio files, reading transcripts thoroughly and taking preliminary notes on recurring ideas and key concepts in the dataset. MM worked closely with the interviewers to ensure accurate reading and interpretation of transcripts. MM coded the first transcript line-by-line to generate an initial set of codes. Codes were reviewed with AW to ensure all relevant concepts in the interview were captured in full. With this insight, the researcher reviewed the initial coding and subsequently coded the remaining transcripts in the dataset. MM and AW regularly discussed coding development, sharing their interpretations of the data and suggesting different ways of approaching the research question. The coding framework was reviewed by MM, AW and CSEH throughout the coding process to facilitate cross-checking, ensure coding consistency across the transcripts and support theme development. The wider research team (CMD, CMH, PM) was also involved in discussions around sub-theme and theme development, emerging findings and interpretation of the results. Themes, subthemes and codes were then organised and iteratively refined to include new insights.

Throughout the analysis process, researchers considered questions of reflexivity by identifying and reflecting on assumptions and preconceptions regarding abortion care. They acknowledged that being strongly in support of reproductive health and rights and having had professional and personal experiences at the ACS influenced their interpretation of the results. They considered these viewpoints as a useful lens through which to engage with and contextualise the dataset but were also mindful of maintaining a non-judgemental attitude to participants’ views and opinions that differed from their own.

### Ethical approvals

Human Research Ethics Approval for the study was obtained from the Royal Women’s Hospital Ethics Committee (Project ID: 79615) and registered with the Monash University Human Research Ethics Committee. Written informed consent was obtained from all participants prior to their interview.

Members of the research team are embedded within the ACS and could potentially be known to participants. To mitigate this, informed written consent was obtained, and participants were assured that participation was voluntary, and that they could withdraw at any time. Participants were given the contact details of a member of the research team for questions and concerns throughout the study.

Supports were made available to researchers in case they experienced any psychological distress while working on the study. The research team had regular fortnightly meetings, providing opportunities for discussion and debriefing, and formal support services (e.g., Employee Assistance Program) at the researchers’ respective institutes were known.

## Results

### Participant characteristics

Overall, 18 providers were invited to participate in our study, of which one declined to participate for reasons undisclosed. In total, 17 health providers participated in the study. The duration of interviews ranged from 25 min to 1 h and 5 min. Most participants were non-Indigenous, Australian-born females who spoke English (see Table 1). The sample included a range of professions including obstetrician-gynaecologists, nurses, midwives, social workers, Aboriginal Liaison Officers, psychiatrists and general practitioners (GP). Participants had varying levels of experience working in any abortion service that ranged from 3 months to 30 years.

**Table 1:** Participant characteristics (*n* = 17)

Characteristic	Description	N
Gender	Female	15
	Male	2
Country of birth	Australia	13
	Overseas	4
Ability to speak a language other than English	Yes	3
	No	14
Identify as Aboriginal and/or Torres Strait Islander	Yes	2
	No	15
Profession	Obstetrician-gynaecologist	5
	Nurse and/or midwife^a^	7
	Social worker	2
	Other	3
Years of experience working in abortion services	0 to 6 months	3
	> 6 months to 12 months	4
	> 1 year to 5 years	3
	> 5 years to 10 years	1
	> 10 years to 20 years	4
	> 20 years	2

^a^Two participants were both a registered nurse and midwife

### Themes

Three themes and nine subthemes were identified (see Table 2). Verbatim quotations are included in italics and numbered according to the interview participant’s identifier (P1-17). Square brackets are included where the authors added words to clarify meaning, and an ellipsis indicates where non-relevant text has been removed for confidentiality, clarity and conciseness.

**Table 2:** Themes and sub-themes

Theme	Subtheme
Being committed to quality care: taking a holistic approach	• Prioritising the woman’s needs • Creating a safe space • Delivering timely care
Surmounting challenges: being an abortion provider is difficult	• Providing a very different kind of abortion • Confronting emotional and ethical challenges • Feeling supported and valued
Meeting external roadblocks: deficiencies in the wider healthcare system	• Strengthening the abortion service • Building capacity in the healthcare system • Struggling through the COVID-19 pandemic

#### Being committed to quality care: taking a holistic approach

Participants spoke of their commitment to providing holistic abortion care by addressing the medical, psychological and social needs of patients attending their service.

##### Prioritising the woman’s needs

Participants prioritised a woman or pregnant person’s needs by engaging an integrated multidisciplinary care team of doctors, nurses, midwives, social workers and an Aboriginal Liaison Officer to provide tailored medical and psychosocial support. For example, providers offer opportunistic medical care, contraception, accommodation and transport support, and counselling to abortion patients. In doing so, *“the needs of the women are always paramount”* (P9). One participant thought that it was *“amazing that [the service] can provide that all under one roof [within one team]”* because *“care in a hospital can be quite fractured”* (P11). Providers also described making referrals to external services where required, such as to mental health services, family violence services, and sexual health clinics. Participants felt that the entire team acted *“with great care and humanity, and [tried] to work in a really patient-centred way that privileges [the patient’s] autonomy and decision making”* (P7). One participant felt that it was important to *“be incredibly flexible, because every woman is so different, and the complexities are so different”* (P9).

##### Creating a safe space

Participants endeavoured to create a safe space for patients at the service, acknowledging the stigma around abortions at 20 weeks and over. One participant commented:

*“They [patients] require a bit of extra support so that they know it’s legal, we’re not judging them, because there is that level of stigma and shame in the community still surrounding abortions. And I think the further along [in pregnancy] you get, the easier it will be to feel that stigma from those around them and from themselves.”* (P8).

Providers at the service described a compassionate, sensitive and non-judgemental approach, as evidenced by one participant who said that staff *“are aligned with them [the patients]. They’re not sitting there wagging their finger, not in the tone of their voice, not in the language that they use…not in any shape or form”* (P17). Another explained their approach: *“it’s about, what does she [the patient] think is right for her? I very strongly believe that’s only for her to know”* (P7). Patients are asked which terminology they would prefer providers to use when referring to the fetus, such as ‘baby’, ‘pregnancy’ or ‘products of conception’. They are also consulted about their intended arrangements for the pregnancy remains, which one participant noted was not always available at other health services. Participants saw it as part of their role to *“ensure that they [patients] had everything they needed to be emotionally okay about the service”* (P9), and to *“help them [patients] to psychologically recover”* (P17) from any internalised guilt they may harbour from seeking an abortion.

##### Delivering timely care

Participants raised the importance of delivering timely abortion care and believed that they largely managed to achieve this at their service. They acknowledged that any delay in accessing an abortion could be *“distressing”* (P1) for a woman or pregnant person and could potentially increase the clinical risk associated with the procedure. However, providers explained that they are *“very good at facilitating quite urgent access [to an abortion]…around the 20-week gestation”* (P3) and responded to most referrals of this kind *“within that day”* (P7). One participant said that for most patients, *“from referral to having their surgery, [it’s] usually within a two-week period”* (P12), despite the ACS being the only service in Victoria providing abortions at 20 weeks and over for psychosocial reasons. Participants felt that *“for the most part, women that need a service, get a service…due to the flexibility of the multidisciplinary team”* (P9). One participant said: *“if this place is full, then we try and make some more room”* (P16). Participants also spoke of there being a *“clear path of support”* (P1) within the service, which is enabled by continuity of care throughout the patient journey. For example:

*“I can follow them [the patients] through from clinic to day surgery to theatre to postoperatively. And so, you can kind of have that journey with the patient. And I think that journey is smooth and supported and caring.”* (P1).

#### Surmounting challenges: being an abortion provider is difficult

Participants felt that being an abortion provider for those requiring this at 20 weeks and over was an immensely challenging role but one that was ultimately very rewarding.

##### Providing a very different kind of abortion

Participants perceived that delivering abortion care at 20 weeks and over was *“quite a different process”* (P8) to providing abortions at an earlier gestation. Many attributed this to the sensitive nature of these abortions: *“a lot of it [the role] is emotional reassurance and counselling aspects of it [as] there’s a huge amount of shame and guilt involved [for the patients]”* (P14). Participants also noted that abortion patients at 20 weeks and over *“have probably more psychosocial complexity”* (P7), such as mental illness and domestic violence, resulting in the need for a sensitive and compassionate approach and for complex and coordinated multidisciplinary care. They also acknowledged that these psychosocial complexities may have delayed their access to abortion services in the first instance. For example:

*“If a woman is in a situation where there’s a lot of psychosocial complexity, like family violence, sexual assault, drug use, instability, they’re all barriers…[to] being able to identify [an abortion] provider, and then having the space to call and participate in all of those conversations in order to get the abortion.”* (P7).

Overall, participants felt that their role necessitated *“high-level assessment skills”* (P9) and a *“depth of understanding about where the women come from psychologically, and the life events that have brought them to see us [the providers]”* (P17).

##### Confronting emotional and ethical challenges

Participants felt that providing abortions at 20 weeks and over was *“a hard job to do”* (P16) and could be very *“mentally and emotionally draining”* (P3). In part, this was attributed to the work being *“high acuity [situations requiring urgent attention]”* (P7) in nature and providers having to manage *“complexity and trauma presentations all the time”* (P7). One participant explains:

*“[Providers are] often taking on board and listening to some really challenging things. Very often you get off a call with someone that [has experienced] sexual assaults in their life and doesn’t have support. So you really have to take on all of this…and take the initiative to get support when you need it.”* (P9).

Some participants acknowledged that this work could be potentially traumatising for staff. For example: *“we still think about patients who we saw one year, two years ago and wonder what happened to them”* (P11). Participants also expressed that performing the abortion procedure could be challenging. One participant said: *“it just is unpleasant. It’s a necessary service that I am absolutely happy to provide but that can be challenging”* (P1).

Participants also discussed the ethical questions that arose while providing abortion at 20 weeks and over for non-medicalised reasons. While some initially found the work confronting, ultimately most participants did not perceive these abortions any differently to those at earlier gestations. For example: *“whether it’s before 20 weeks or after it’s exactly the same, I think it’s whatever’s best for them [the patient]”* (P13). Some participants did perceive abortions at 20 weeks and over for non-medicalised reasons to be ethically challenging, particularly if they had worked in obstetric services. One participant said: *“I do morphology ultrasounds*^3^*at 20 weeks for wanted pregnancies, and we deliver wanted pregnancies at 23 weeks and resuscitate the baby, so 20 weeks feels like a like a big threshold to go over”* (P16). However, participants felt supported to *“express [their] bounds and limits”* (P3) if there was *“something [they didn’t] want to be a part of”* (P3).

##### Feeling supported and valued

Participants felt very supported in their role at the ACS due to the *“good relationships”* (P7) in their *“open and tight knit”* (P11) team and the *“cohesive working environment”* (P6) that this created. They remarked that their peers were *“very generous around checking in and sharing workload”* (P7) and they felt comfortable seeking help from managers, whom one participant described as *“supportive and responsive”* (P7). They valued having regular team meetings and clinical supervision and were aware that the hospital’s Employee Assistance Program and private psychology services were available if required. Participants felt *“very valued”* (P7) in their role as an abortion provider and found it to be *“an incredibly satisfying area of women’s health to be a part of”* (P3). They felt rewarded by being able to provide a *“life-changing”* (P1) and *“essential”* (P10) service to women and pregnant people that aligned with their personal ethos of supporting reproductive rights and autonomy. They also took pride in delivering what they perceived to be high quality abortion care. For example: *“We’re [providers] all there for the same reason. We all genuinely want to help our patients. And I think we do provide really exceptional patient care”* (P17).

#### Meeting external roadblocks: deficiencies in the wider healthcare system

Participants noted that systemic issues in the healthcare system, such as the limited abortion workforce, had a negative downstream effect, creating external roadblocks to their ability to deliver a quality abortion service.

##### Strengthening the abortion service

Participants identified that their abortion service could be strengthened by building capacity in the workforce, as many felt that the ACS was understaffed. This was in line with observations that abortion was generally an *“underserviced field”* (P9). One participant remarked that the workload at the service was *“just relentless…the phone’s always ringing, there’s always something to follow up on”* (P9). Participants felt that the service was *“not anywhere near as resourced as [it] should be”* (P7). Understaffing could also limit the time available for debriefing and professional development opportunities, which in turn impacted staff retention and workforce sustainability. For example: *“we think of best care involving opportunities for research and training and things that can add to us developing and upskilling. There just isn’t as much time for that”* (P9). This had flow-on effects for patient care, such as midwives being unable to always provide one-to-one aftercare^4^ once a patient having an induced abortion had birthed, and the service being unable to *“facilitate a full spectrum of [follow-up] care”* (P9), including medical and psychosocial support, once a patient had been discharged.

##### Building capacity in the healthcare system

Participants highlighted the urgent need to build capacity in the healthcare system for the delivery of abortion at 20 weeks and over for psychosocial reasons. Many discussed the *“enormous pressure”* (P10) on the ACS as the *“sole provider [of these abortions] for all of Victoria”* (P9) and the implications that this had for equitable access to care, particularly for people living regionally and rurally. For example: *“if you’re in the country, where do you go? We don’t have any providers [there] who are skilled at doing these [abortion procedures]”* (P1). Participants urged the *“expansion of public provision of abortion”* (P12) in Victoria and suggested that state government policies should be implemented to achieve this. Many felt that the pervasive stigma surrounding abortion at 20 weeks and over was a significant factor underlying the paucity of services. One participant remarked: *“obstetricians and gynaecologists are [still] not as a whole comfortable with [abortion]”* (P11), and it was suggested that there are limited opportunities for obstetrician-gynaecologists to train in abortion surgeries. The same participant also said:

*“The idea that women over 20 weeks or 24 weeks would consider termination, it doesn’t get talked about in the community, doesn’t get talked about in your training. And so it just fades into the background.”* (P11).

Another provider expressed that a *“really big cultural shift”* (P7) that involved *“more openly acknowledging sexual and reproductive health [as a whole]”* (P7) was needed to substantially address the issue.

##### Struggling through the COVID-19 pandemic

Despite abortion being considered an essential service during the COVID-19 pandemic emergency, participants felt that service provision was impacted considerably, which exacerbated pre-existing challenges in their role. Staff being furloughed and taking sick leave due to either having COVID-19 or being a close contact in the first years of the pandemic *“very much impacted on a small service”* (P10) that is already *“running on skeleton staff”* (P10) and meant that abortions sometimes had to be delayed. The administrative burden on staff increased substantially, due to having to organise visitor exemptions and check vaccination status and test results, which *“took up a lot of clinical [time]”* (P7). At times, visitor restrictions meant that staff had to step in to be the patient’s social support. Participants felt this negatively impacted the patient experience: *“she’s [the patient] talked to us on the phone once [and] never met us in person, and we are the support. That’s not ideal by any means”* (P9). Some participants noted that having to wear PPE was *“challenging”* (P16) in such an *“emotionally charged”* (P16) clinical environment and affected the *“interpersonal element”* (P5) of care.

Participants also observed a dramatic increase in the number of patients presenting for an abortion at 20 weeks and over during the pandemic. They hypothesised this to be due to a delay in accessing primary care services, resulting in a delayed pregnancy diagnosis and referral to an abortion service, which was seen to be already *“challenging in a ‘normal’ world”* (P9). One participant noted that difficult psychosocial circumstances were exacerbated in the pandemic, amplifying the barriers to accessing an abortion. They said:

*“In the midst of COVID, with kids who are homeschooling, with a partner who’s home all the time, but doesn’t know she’s pregnant, and she’s trying to conceal that. There’s so many of those difficult social situations which were just aggravated in COVID and therefore the barrier just got bigger for that woman to come see us.”* (P11).

A reduction in the private provision of abortion during the pandemic was also thought to contribute.

## Discussion

We set out to examine health providers’ perceptions and experiences of providing abortion care at 20 weeks and over for indications other than fetal or maternal medicine, as well as enablers and barriers to this care and how quality of care could be improved in one hospital in Victoria, Australia. We found that providers in our study were committed to delivering holistic abortion care that centred women and pregnant people’s needs and autonomy. However, at times they could feel emotionally overwhelmed and challenged by ethical questions that arose in their role. Providers also observed that the lack of abortion services at 20 weeks and over in Victoria compromised equitable access to care and they identified the COVID-19 pandemic as a serious barrier to delivering timely care.

The World Health Organization defines quality abortion care as being effective, efficient, accessible, acceptable (person-centred), equitable and safe [1]. There should be information provision and counselling, where desired by the patient, and care should be centred around patients’ values and preferences [1]. Indeed, participants in our study made every effort to prioritise patients’ needs, minimise stigma, and provide a safe and timely service, despite staffing constraints. There is little research that describes what constitutes quality abortion care at 20 weeks and over, which is unique due to the psychosocial and medical complexities at this gestation. Participants in our study felt that consulting patients on their intended arrangements for the pregnancy remains, and providing comprehensive psychosocial care, were particularly important aspects of care at this gestation.

Providers of abortion at all gestations have been found to face many challenges in their role, including grappling with the ethical considerations of their work, and at times experiencing negative emotional impacts, such as anxiety, sadness and grief [17–20]. Many have described having inadequate supports in place to manage these challenges [17, 20, 21]. Though providers in this study similarly found their work difficult at times, they highlighted that the supportive team environment enabled them to successfully navigate emotional and ethical challenges. Participants felt that they could rely on colleagues and managers for support, with whom they had good relationships, and found structured supportive supervision such as team meetings and clinical supervision to be a helpful tool for navigating difficulties that could arise. Our study suggests that these may be vital elements to supporting abortion providers’ wellbeing and promoting satisfaction in their role. Many studies have found that providers engage in abortion work due to personal beliefs and values around supporting reproductive autonomy and therefore find their work rewarding [17, 18, 22], which was mirrored in our study. This may be a protective factor in sustaining providers in their challenging role.

Workforce shortages exist at all levels of abortion provision in Victoria, limiting the availability of services and impacting equitable access to care [12, 23]. This has been attributed to difficulty attracting providers to work in abortion services due to stigma, conscientious objection, and limited training opportunities for medical students and obstetrician-gynaecologist trainees and other staff [24]. Our research highlights workforce limitations as a significant barrier to being able to provide the service. People living in rural and regional areas are particularly impacted, as they face additional challenges to abortion access such as a limited availability of GP appointments, poor information provision and conscientious objection by GPs, stigma, cost and transport barriers, and concerns about confidentiality [24–27]. A decline in private services providing abortion at 20 weeks and over has been observed in recent years, although the reasons for this are unclear [12]. The reproductive health and rights of women and pregnant people are threatened as a result. Leadership and culture have been identified by the WHO as key components of an enabling environment for abortion care [1]. Healthcare leadership can contribute to legislation and institutional policies and environments that are supportive of abortion provision, such as commitments to building a sustainable workforce and enhancing public provision [28–30]. Participants in our study spoke of supportive leadership from managers and a strong commitment to reproductive rights as key enablers of quality abortion provision at their service. Fostering leadership and a culture that support and enable abortion provision is critical in building capacity for abortion provision at 20 weeks and over in the healthcare system.

The COVID-19 pandemic emergency caused significant disruptions to the delivery of sexual and reproductive healthcare worldwide [31, 32]. The interviews in this study were conducted in 2022 with thousands of COVID-19 cases in the Victorian community, but disease control measures had eased significantly compared to 2020 and 2021, which saw strict lockdowns and movement restrictions [33]. Participants described barriers to delivering abortion care during the pandemic including visitor restrictions, increased administrative requirements, and understaffing, similarly reported to have impacted abortion services globally [34, 35]. While the COVID-19 pandemic prompted innovations to maintain abortion accessibility in some contexts, such as the provision of early medical abortion via telehealth [32], participants in our study did not observe these changes at their service. Instead, they thought that the pandemic delayed access to their service and saw more patients presenting at 20 weeks and over, necessitating a more complex and difficult procedure than at earlier gestations.

### Strengths and limitations

A major strength of our study was that it explored the novel perspectives of abortion providers at 20 weeks and over for psychosocial reasons in Victoria, Australia, the first study of its kind in the Australian context. Additionally, a diverse range of professions and levels of experience were captured in the sample. Our study was limited by participants being mostly non-Indigenous, Australian-born, English-speaking females, meaning that perspectives outside of these demographic parameters may not have been captured. The ACS also has strong institutional support for its work, a significant enabler to abortion provision, and findings may differ in settings where this is not the case. Our study may have been impacted by social desirability bias, whereby participants give responses that they believe the interviewers want to hear, rather than their true opinions or experiences, due to interviewers also being ACS staff members. However, the study was led by ACS staff together with an independent medical research institute to mitigate these issues. Whilst this paper did not report on service user experiences of abortion care, this work is part of a larger study including exploring user experiences of abortions at 20 weeks and over, which will complement this work and give unique and important insight into user experiences of quality of care.

### Implications for policy and practice

The findings point to an urgent need for more services providing abortion at 20 weeks and over for non-medicalised reasons in Victoria, to secure access to safe and equitable care. A state-wide abortion strategy that outlines adequate service provision, particularly in public hospitals, supports training and workforce development and addresses stigma would be an important step in this process. More research investigating the care experiences of abortion at 20 weeks and over for psychosocial reasons from provider and user perspectives throughout Australia and worldwide is needed to compare findings between contexts. Furthermore, research that evaluates strategies to strengthen the abortion workforce, investigates stigma-reduction measures and engages and encourages hospitals to provide affordable, accessible and acceptable care, is urgently needed.

## Conclusion

Abortions at 20 weeks and over for indications other than fetal or maternal medicine are highly stigmatised and often associated with significant access difficulties. The procedure involved is more complicated compared to abortions at an earlier gestation and can be challenging for both patients and providers. Providers in our study felt well supported at their service to provide holistic, person-centred care and confront the emotional and ethical challenges of their role, but workforce gaps in the abortion field were felt to limit their service’s capacity and negatively impact equitable access to safe and timely care. There is a need for supportive policies and frameworks to strengthen the abortion workforce and expand provision of affordable, acceptable and accessible abortions at 20 weeks and over in Victoria, Australia.

### Electronic supplementary material

Below is the link to the electronic supplementary material.

Supplementary Material 1

## Data Availability

The datasets generated and analysed during the current study are not publicly available due to confidentiality issues but are available from the corresponding author on reasonable request.

## Footnotes

1 In our study we use the term ‘woman or pregnant person’ to recognise that those who are pregnant may identify as a pregnant woman, a pregnant person or both. We use this language to be inclusive to all people who may identify as such.

2 Abortion for indications other than fetal or maternal medicine (also referred to as ‘psychosocial’ or ‘non-medicalised’ indications) is defined as an abortion sought for reasons relating to the psychosocial conditions in a woman or pregnant person’s life, such as economic disadvantage, sexual assault and family violence, and excludes abortion sought for reasons of fetal abnormality and maternal health condition.

3 Morphology ultrasounds are a routine antenatal test undertaken at around 20 weeks’ gestation to assess the fetus’s growth and development.

4 Aftercare for a patient undergoing an induced abortion in birth suite can include creating a memory folder (of the baby’s photographs, handprints and footprints, weight etc.) and having time with the baby, if the patient wishes.

## Abbreviations

ACS: Abortion and Contraception Service
GP: General Practitioner
SDGs: Sustainable Development Goals
