# Supplementary material for: Abortion care at 20 weeks and over in Victoria: a thematic analysis of healthcare providers’ experiences
Source: BMC Pregnancy Childbirth. 2024 Feb 6;24:112. doi: 10.1186/s12884-024-06299-0 (PMC10845525; doi:10.1186/s12884-024-06299-0)
Supplement: Supplementary file 1 — Supplementary Material 1 [file 12884_2024_6299_MOESM1_ESM.docx]

# Additional file 1. Interview guide

## Demographic/background questions

Ok, the first thing I’d like to ask are some background and demographic questions.

1. What is your profession? (Doctor, nurse, social worker etc.)
2. How long have you been working in abortion services?
3. Which of the following best describes your current gender identity?
   Male, Female, non-binary/gender fluid, or other? (Please specify)
4. Do you identify as Aboriginal and/or Torres Strait Islander?
5. Were you born in Australia or overseas?
   1. If yes, where?
6. Do you speak any languages other than English?
   1. [If yes] What are they?

## Interview questions

1. Can you tell me a bit about what your current role involves within abortion care unit? (e.g., support services, counselling, medical review etc)

Prompt: How long have you been working in your current role and providing these services?

1. Can you tell me about your general experiences in providing these services?
2. How often do you work with people seeking abortions at or after 20 weeks?
   1. Can you tell me about how these patients are referred to you and the abortion service you work at?
      1. Are these referral pathways efficient? Are these pathways easy for patients and clinicians to navigate? Why/Why not?
      2. Are referrals always appropriate? Why/Why not?
      3. Are referrals always timely? Why/Why not?
   2. What additional care/support do you need to provide to those seeking abortions at or over 20 weeks? Can you tell me about this?
3. Do you perceive any additional ethical considerations when you are providing care to those who are at or over 20 weeks compared to those who present for abortions earlier?
4. Do you feel adequately supported as a health practitioner to provide these services?
5. Medical review boards are used in cases over 24 weeks. Can you tell me how you feel about the use of these boards?
   1. Are there any positive or negative effects of using ethics boards?
6. Accessing abortion services are time sensitive, do you feel that your service timely access to abortions at or over 20 weeks?
   1. Why/why not, are there any barriers, hold-ups to providing services?
7. What do you think your service does really well when providing abortions services at or over 20 weeks?
8. What do you think is currently lacking in providing services to those seeking abortions at this time?
9. In your opinion how can services for those presenting after at or over 20 weeks be improved?
   1. What would need to be done to achieve that?
10. Do you think there are any barriers to providing quality care within your scope of practice within the abortion care unit?
    1. Prompt: Can you describe these barriers?
    2. How could these barriers be overcome?
11. Do you feel all people who require abortions at or over 20 weeks in Victoria are able to access services? Why/Why not? Are there any specific population groups that might be missed in the current provision of services? How can that be improved?
12. Do you ever refer people seeking abortions at or over 20 weeks to other services, this can include support services, either during or after admission?
    1. Can you describe these services?
    2. Are there sufficient services to refer to or are there gaps in other services needed? Can you tell me more about this?
    3. Can you describe your reasons for referring on to other services?
13. Do you feel you have enough access to resources, links and other pieces of take-away information to give to those who access abortions at or over 20 weeks?
    1. Can you tell me more about this? What else is needed?
14. Do you think those who access abortions at or over 20 weeks feel supported by the current level of services provided to them?
    1. Why/ Why not? Can you tell me more about this?
15. Has your specific role in providing services to those seeking abortions at or over 20 weeks been impacted by the COVID-19 pandemic?
    1. If yes – how?
    2. Have any at or over 20 week abortion services been provided over telehealth? How did this work? Is there a place for ongoing telehealth appointments in over 20 week abortions?
16. What are your recommendations for key services that must be available to people seeking abortion services at or over 20 weeks?
17. Are there any internal or state-wide policies that need to change to optimise the current provision of services?
18. Is there anything else you would like to tell me about delivering services to those seeking abortions at and over 20 weeks?
